# Impact of Surgical Timing and Approaches to Health Care Utilization in Patients Undergoing Surgery for Acute Traumatic Cervical Spinal Cord Injury

**DOI:** 10.7759/cureus.6166

**Published:** 2019-11-15

**Authors:** Mayur Sharma, Nicholas Dietz, Beatrice Ugiliweneza, Dengzhi Wang, Nicolas K Khattar, Shawn W Adams, Tyler Ball, Maxwell Boakye

**Affiliations:** 1 Neurosurgery, University of Louisville School of Medicine, Louisville, USA

**Keywords:** anterior, posterior, circumferential, cervical spine fusion, spinal cord injury

## Abstract

Objective

Acute traumatic cervical spinal cord injury (AcSCI) causes significant morbidity and has an impact on health care utilization. The aim of our study was to analyze health care utilization in patients undergoing surgical decompression and fusion for AcSCI based on timing and type of surgical approaches.

Patient and methods

Data were extracted using ICD9/10 and CPT codes from MarketScan (IBM Corp. Armonk, New York [v. 2000-2015]). We defined the comparative groups based on the timing of surgery (early <24 hours and late >24 hours) and surgical approaches: anterior, posterior and circumferential. Outcomes of interest were: length of hospital stay, discharge disposition and health care utilization in the index hospitalization, within 30 days after discharge and 12 months following injury.

Results

Of 1604 patients, 80.9% had early procedures and 55.7% of these had anterior-only procedures. Overall, the median age was 46 years in the early surgery group and 47 years in the late surgery group. Patients in the early surgical group incurred higher outpatient services and there was no difference in cumulative median payments (index + 12 months) across the cohorts (early: $127,379, late: $121,049). The incidence of repeat surgery at the index level did not differ based on the timing of surgery (early 5% vs. late 7%). Complications were higher in the circumferential surgery cohort irrespective of the timing of surgery. Overall, combined median payment (index hospitalization + 12 months) was significantly higher for early circumferential cohorts compared to the anterior or posterior-only cohort ($195,990 and $109,977 vs. $121,236 respectively).

Conclusion

Late (>24 hours) surgeries were associated with a higher likelihood to be discharged home, lower utilization of outpatient services, higher hospital readmissions and no differences in payments (index and cumulative) compared to early surgeries. Circumferential approaches were associated with higher complication rates, lesser likelihood to be discharged home, higher utilization of outpatient services compared to anterior-only approaches.

## Introduction

Acute traumatic cervical spinal cord injury (AcSCI) represents a devastating event that causes significant morbidity and mortality, and has an annual incidence of 15-83 per million worldwide [1-2]. Further, prevalence is highest in the United States with 906 cases per million people annually [2]. AcSCI constitutes up to 70% of all traumatic spinal cord injuries [3]. Leading etiologies of SCI include motor vehicle accidents, falls, violent acts, and contact sports [4]. Health care costs stem from greater payments required for acute care in intensive care unit facilities, higher rates of complications related to the injury, rehabilitation and health services [5]. 

Diverse adverse health outcomes often follow SCI, including negative effects on multiple organ systems such as musculoskeletal, renal, digestive, cardiovascular and respiratory systems [6-7]. The National SCI database demonstrated that quadriplegics outnumber paraplegics as the most common subset of injury patients and have life expectancies that exceed 30 years [4]. Krueger and colleagues reported that the lifetime economic burden of SCI in Canada for incomplete paraplegia is around $1.47 million while that for complete tetraplegia is approximately $3.03 million [8]. Additionally, the economic burden of 1,389 individuals amounted to $2.76 billion annually. 

Based on a recent trial (2017) addressing Surgical Timing in Acute Spinal Cord Injury Study (STASCIS), most of the surgical decompressions are performed within 24 hours of injury [9-10]. Also, early surgery has been shown to be associated with decreased health care utilization and length of hospital stay in patients with acute SCI [11]. The present study represents a comprehensive analysis of health care utilization in patients undergoing surgical decompression and fusion for AcSCI, using the MarketScan (IBM Corp. Armonk, New York) database. Specifically, we describe index hospitalizations, hospital readmission, medications, and rehabilitation services up to 12 months after injury. We stratified the patients according to the timing of surgery (early <24 hrs and late >24 hrs) and surgical approaches for AcSCI as anterior, posterior and circumferential. We hypothesize that patients who had circumferential fusion and late surgeries for AcSCI incurred higher costs compared to other procedures at the index hospitalization and at 12 months. We also aimed to highlight the differences between only anterior and posterior approaches in terms of complications and health care utilization. A greater understanding of the pathophysiology of SCI and the proper management of this affliction will continue to change the economic impact and individual payments associated with traumatic spinal cord injury. To the best of our knowledge, this is the first study focusing on the timing and impact of the types of surgical approaches on health care utilization in this patient cohort using a national administrative database.

## Materials and methods

Data source

In this retrospective study, we used MarketScan data from Truven Health Analytics - IBM Watson Health as described previously [12-13]. Included in this database are paid claims by employer-sponsored insurance, COBRA, private-insurance-managed Medicaid and Medicare Supplemental (also known as Medigap). More than 100 payers contributed to this data. Individuals enter the data when they enroll with their insurance and exit when that coverage ends. These individuals are the primary insurance holders or their dependents. While enrolled, their continuum of care is captured along with the corresponding payments. The data is de-identified. Each individual has a unique encrypted ID that can be used to link to different databases [14]. Researchers have used this database for medical, public health and epidemiology research for decades [14]. We have a custom database of neurological /neurosurgical conditions spanning 16 years (2000-2016) across three insurance types (private, Medicaid and Medicare supplemental) covering inpatient admissions, outpatient services and outpatient prescription medications. We used this data to extract patients who suffered traumatic AcSCI and underwent surgery using International Classification of Disease (ICD) 9/10 and Current Procedural Terminology (CPT) codes.

Patient selection and follow up

From the inpatient tables, we extracted patients with a diagnosis of AcSCI. An inclusion criterion was decompression (laminectomy, laminotomy, discectomy, vertebrectomy, corpectomy, foreign body removal and repair of vertebral fracture) with concurrent fusion. We created three analysis groups based on the fusion approach: anterior, posterior and circumferential (concurrent anterior and posterior). The claim codes used for extraction are in (Table 1). Pediatric cases (patients under 18 years old) were excluded. For each patient, the first-occurring case satisfying the above conditions was flagged as the index hospitalization. To make sure that this was the time of injury, all patients retained had to be continuously enrolled for the previous six months without any claim (inpatient or outpatient) of spinal cord injury (traumatic or non-traumatic, at any level of the vertebral column). We were interested in the outcomes and health care utilization in the 12 months following injury; therefore, only patients who had 12 months post-surgery continuous enrollment in their insurance were included in the analysis data set. To calculate the look-back period and the follow-up time, we used the dates of start and end enrollment times: pre-diagnosis look-back time = surgery admission date - start enrollment date (or first claim date in the data set); post-surgery follow up time = end enrollment date (or last claim date in the data set) - surgery discharge date.

**Table 1:** Table showing ICD-9/10 codes used for data extraction

	ICD-9	ICD-10	CPT
Cervical spinal fusion	806.0, 806.1	Initial encounter S12 or S17 with concurrent S141	
Cervical Decompression	03.01 03.09 03.4 03.53	00CW4ZZ, 00CW4ZZ, 00CW0ZZ, 0RG13ZZ, 0RB10ZZ, 0RB04ZZ, 00NM4ZZ, 00NW3ZZ, 00NW0ZZ, 00BW0ZZ, 00BW3ZZ, 00BW4ZZ, 005X0ZZ, 005X3ZZ, 005X4ZZ, 0PS30ZZ' 0PS334Z, 0PS33ZZ, 0PS344Z, 0PS34ZZ, 0PS3XZZ	63001, 63015, 63020, 63040 63045, 63050, 63051, 63075 63081, 63180, 63194, 63196 63198, 63250, 63265, 63270 63300, 63304
Anterior fusion	81.02	0RG1070, 0RG107J, 0RG10A0, 0RG10AJ, 0RG10J0, 0RG10K0, 0RG10Z0, 0RG10ZJ, 0RG1370, 0RG13J0, 0RG13K0, 0RG13Z0, 0RG1470, 0RG14A0, 0RG14J0, 0RG14K0, 0RG14Z0, 0RG2070, 0RG20A0, 0RG20J0, 0RG20K0, 0RG20Z0, 0RG2370, 0RG2371, 0RG23A0, 0RG23J0, 0RG23K0, 0RG23Z0, 0RG2470, 0RG247J, 0RG24A0, 0RG24J0, 0RG24K0, 0RG24Z0	22551, 22554
Posterior fusion	81.03	0RG1071, 0RG107J, 0RG10A1, 0RG10AJ, 0RG10J1, 0RG10JJ, 0RG10K1, 0RG10KJ, 0RG10Z1, 0RG10ZJ, 0RG1371, 0RG137J, 0RG13A1, 0RG13AJ, 0RG13J1, 0RG13JJ, 0RG13K1, 0RG13KJ, 0RG13Z1, 0RG1471, 0RG147J, 0RG14A1, 0RG14AJ, 0RG14J1, 0RG14JJ, 0RG14K1, 0RG14KJ, 0RG14Z1, 0RG14ZJ, 0RG2071, 0RG207J, 0RG20A1, 0RG20J1, 0RG20JJ, 0RG20K1, 0RG20KJ, 0RG20Z1, 0RG2371, 0RG23A1, 0RG23J1, 0RG23JJ, 0RG23K1, 0RG23KJ, 0RG23Z1, 0RG2471, 0RG24A1, 0RG24AJ, 0RG24J1, 0RG24JJ, 0RG24K1, 0RG24KJ, 0RG24Z1	22590, 22595, 22600

Analysis groups and patient characteristics

We defined the comparative groups based on the timing of surgery (early <24 hrs and late > 24 hrs), and also the fusion approach: anterior, posterior and circumferential.

The following patient characteristics were noted at the time of index hospitalization: age, gender, year of index hospitalization, insurance type (commercial, Medicaid, Medicare) and comorbidities. Comorbidities were measured with the Elixhauser comorbidity score [15] computed using an adaptation to ICD-9-CM codes developed by Quan et al. [16]. These were summarized and included in analyses.

Post-surgery outcome variables

We were interested in outcomes and utilization in the index hospitalization, within 30-days after discharge and within the acute phase of SCI (12 months following injury) as well as the associated payments. 

Complications

We considered the following complications: renal, cardiac, deep vein thrombosis or pulmonary embolism (DVT/PE), pulmonary, infection, wound infection, pneumonia, myocardial infarction, acute kidney injury, pressure ulcers and sepsis as well as general neurological complications. The ICD-9 codes used to search for complications are mentioned in Table 2. The incidence of complications was evaluated during the index hospitalization, and within 30 days after discharge. The presence of any complications were noted as the occurrence of any of the complication types described above. 

**Table 2:** Claim codes for complications DVT/PE: deep-vein thrombosis or pulmonary embolism

Complication	ICD-9	ICD-10
Renal	583, 9975	N17, N9989
Cardiac	410, 9971	I21, I977, I978
Neurosurgical	9970, G97	9970, G97
Neurological	4382, 4383, 4384, 4385	I60, I61, I62, I63, I64, I65, I66, I67, I68, I69
DVT/PE	415, 451, 452, 453	I26, I80, I81, I82
Pulmonary	5184, 5185, 5188, 9973	J810, J80, J951, J952, J953, J958, J96
Infection	5950, 5959, 5990	N3000, N3001, N3090, N3091, N390
Wound infection	99832, 99851, 9986, 99881, 99883	T8131, T814, T818
Pneumonia	481, 482, 486	J13, J14, J15, J16, J17, J18
Myocardial infarction	410	I21
Acute kidney injury	584	N17
Pressure ulcers	707	L89
Sepsis	99591, 99592	A4181, A419, R652

Health care resources use

Our interest was to look at the index hospitalization as well as the post-discharge health care resources use during the acute phase of the injury. For the index hospitalization, we analyzed the length of hospital stay (days from hospitalization admission to discharge) and discharge disposition. For post-discharge health resource-use outcomes, we focused on 30-day hospital re-admission and emergency room (ER) admissions, and on 12-month hospital admissions, outpatient services and medication refills. 

Health care resources payment

All the payments associated with the health care utilization described above were analyzed: index hospitalization payment, post-discharge inpatient, and outpatient and medication payments. Payments were the sum of all hospitalizations (inpatient payments), all outpatient services (outpatient payments) and all prescription medication refills (medication payments). We also looked at a combination of all three. ER services are a subset of the inpatient admissions or outpatient services and therefore they were not added in the summation. All payments were inflated to 2016 US dollars using the medical component of the consumer price index which can be accessed through the United States Bureau of Labor Statistics website [17]. 

Statistical analysis 

We summarized continuous variables using means and standard deviations, median and interquartile range as well as the full range (minimum to maximum). Categorical variables were summarized using counts and percentages. To compare the patient characteristics and outcomes among different fusion approaches, we used the Kruskal Wallis test for continuous variables and the chi-square test for categorical variables [18]. We also performed a multivariable analysis for each outcome using linear regression on log-transformed values for continuous outcomes and logistic regression for categorical outcomes. In these models, we included all patient characteristics (gender, age, Elixhauser index and insurance type [19]) in addition to the fusion approach. From these models, we built a linear contrast to compare different groups to the reference group. Adjusted comparisons were presented in terms of estimate ratio (ER) for continuous variables and odds ratio (OR) for categorical variables with associated 95% confidence intervals (CI) as a measure of precision. We used the software SAS 9.4M5 (SAS Institute, Inc, Cary, North Carolina) for data pre-processing and data analysis [20].

## Results

**Figure 1:**
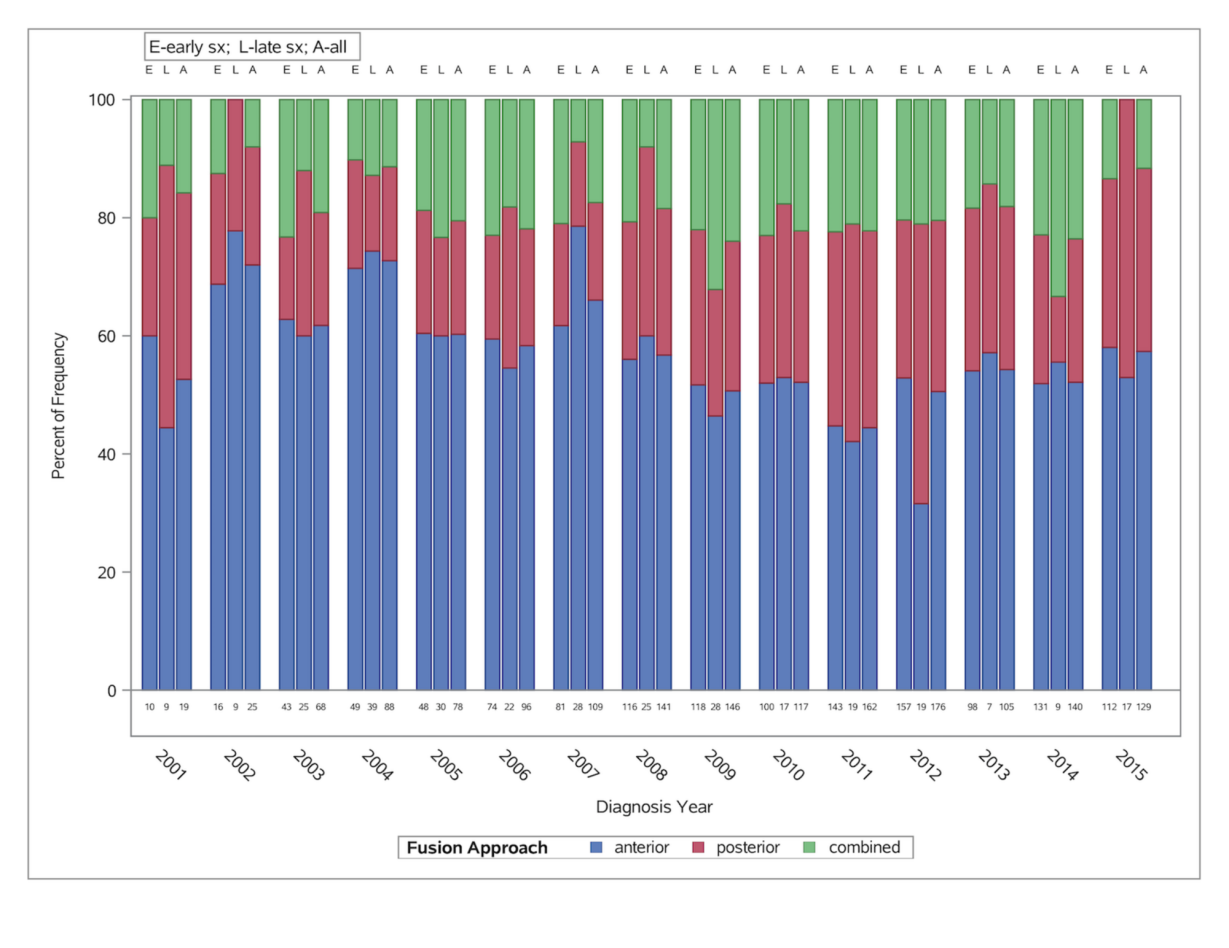
Bar graph showing the trends (2001-2015) of different surgical approaches and timing of surgery (early vs. late) in patients with traumatic acute cervical spinal cord injury

**Figure 2:**
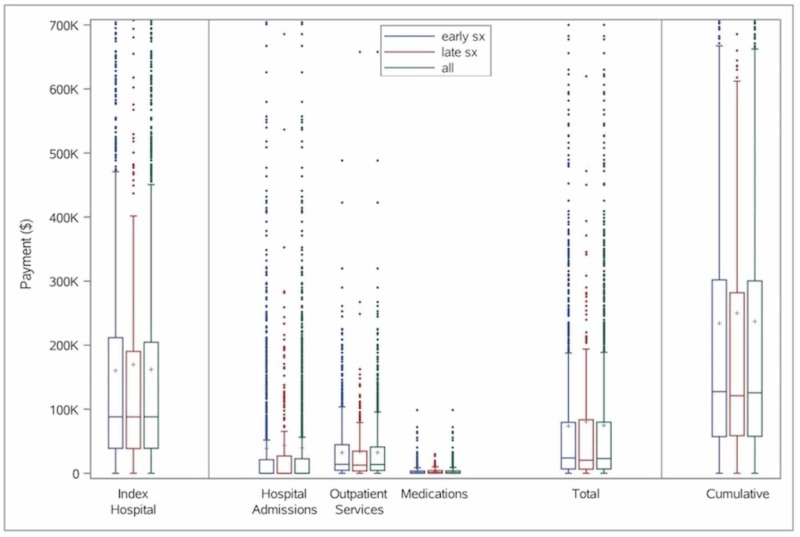
Bar graph showing total payments (from injury to 12 months) based on timing of surgery in patients with traumatic acute cervical spinal cord injury, from 2001-2015

**Figure 3:**
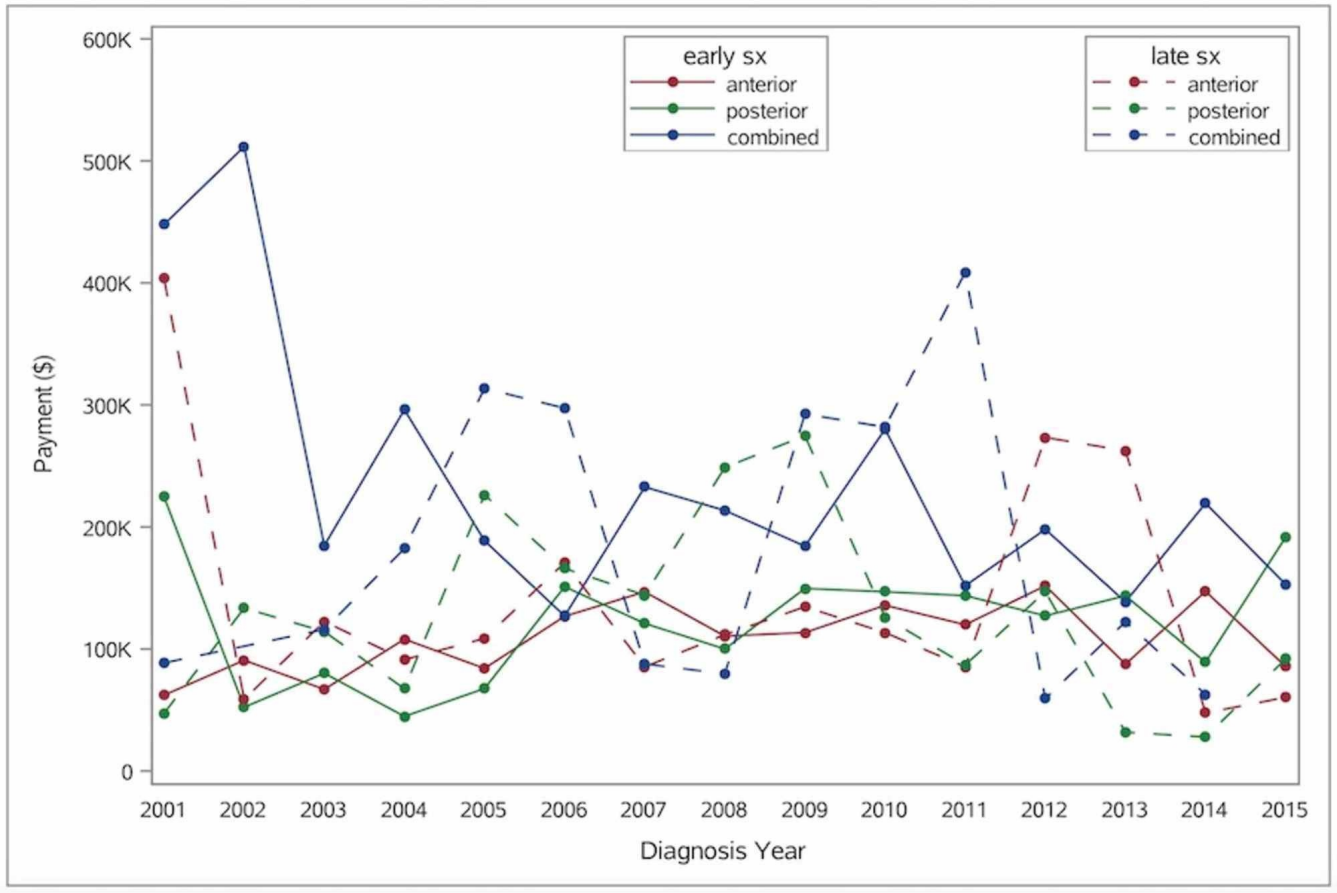
Graph showing total payments based on type (anterior vs. posterior vs. combined) and timing (early vs. late) of surgical approaches (2001-2015) in patients with traumatic acute cervical spinal cord injury

Overall, 1,604 patients underwent decompression and fusion (with six months pre-injury and 12 months post-injury follow-up) for AcSCI during the study period and were thus included in the analysis. Posterior cervical decompression and fusion for AcSCI are increasingly being performed in recent years, whereas the incidence of anterior procedures has remained stable over the last few years (Figure 1). The median 12-months cumulative payments have remained stable from 2001 to 2015 with peak in 2011 for late circumferential procedures. Total payments (from injury to 12 months) based on the timing of surgery and across different surgical approaches over the years are shown below (Figures 2-3).

Patient demographics

Of 1604 patients, 80.9% had early procedures (< 24 hrs); 55.7% of these had anterior-only procedures, followed by posterior-only (25%) and circumferential procedures (19.3%). Among patients with late procedures, anterior-only constituted 58.4% of procedures, followed by posterior-only (25.9%) and circumferential procedures (15.7%) (Table 3). Overall, median age was 46 years (IQR: 28-58 years) in the early surgery group and 47 years (IQR: 31-59 years) in the late surgery group. Patients were younger in the anterior-only group [44 (early) and 46 (late) years] compared to those in posterior-only cohort (52 and 53 years) p < 0.05. A majority of patients were males (68% and 61%) with no difference across the cohorts. A majority of patients with AcSCI had commercial insurance (67% and 59%) and majority of these patients with commercial insurance had anterior procedure, p<0.05. Only a minority of patients had Elixhauser comorbidity index of 3+ in both early (18%) and late surgery (20%) cohorts (Table 3).

**Table 3:** Early and Late surgery characteristics of the sample.

		Early total	Early Fusion				Late total n=305	Late fusion				Early vs. late Sx total, p
			Anterior	Posterior	Circumferential			Anterior	Posterior	Circumferential		
Demographics		n = 1299	n = 715	n = 322	n = 262	p		n = 178	n = 79	n = 48	p	
	Mean (SD)	45 (19)	43 (18)	50 (19)	43 (19)		46 (19)	44 (18)	52 (19)	43 (17)		
Age	Median (IQR)	46 (28-58)	44 (27-56)	52 (35-63)	43 (24-58)	<0.0001	47 (31, 59)	46 (28, 56)	53 (38, 64)	44 (29, 55)	0.0036	0.1778
	Range, min-max	18-92	18-91	18-92	18-89		18-90	18-90	18-89	18-77		
Gender: Female, n (%)		419 (32%)	217 (30%)	119 (37%)	83 (32%)	0.1062	119 (39%)	72 (40%)	32 (41%)	15 (31%)	0.4857	0.0792
	Commercial, n (%)	865(67%)	504 (70%)	187 (57%)	174 (66%)		181 (59%)	111 (62%)	42 (53%)	28 (58%)		
Insurance	Medicaid, n (%)	264 (20%)	139 (19%)	66 (21%)	59 (23%)	<0.0001	83 (27%)	49 (28%)	18 (23%)	16 (33%)	0.0261	0.036
	Medicare, n (%)	170 (13%)	72 (10%)	69 (21%)	29 (11%)		41 (13%)	18 (10%)	19 (24%)	4 (8%)		
	0, n (%)	360 (28%)	214 (30%)	85 (26%)	61 (23%)		89 (29%)	58 (33%)	20 (25%)	11 (23%)		
Elixhauser	1, n (%)	411 (32%)	237 (33%)	97 (30%)	77 (31%)	0.0472	85 (28%)	47 (26%)	25 (32%)	13 (27%)	0.4762	0.6946
index	2, n (%)	297 (23%)	157 (22%)	75 (23%)	65 (25%)		71 (23%)	44 (25%)	16 (20%)	11 (23%)		
	3+, n (%)	231 (18%)	107 (15%)	65 (20%)	59 (23%)		60 (20%)	29 (16%)	18 (23%)	13 (27%)		

Outcomes at index hospitalization, 30-days and 12-months post-discharge

Early vs. late surgery

Median length of hospital stay (LOS) was longer following late surgery compared to those who underwent early surgery (nine days vs. seven days, p < 0.0001). Interestingly, patients were less likely to be discharged home (47%) following early surgery compared to the late surgery cohort (55%), p=0.016. There was no difference in terms of complications during index hospitalization or 30 days post-discharge based on the timing of surgery. Although patients in early surgical group incurred higher outpatient services and corresponding median payments (early: $ 13,801, late: $12,635), there was no difference in cumulative median payments (index + 12 months) across the cohorts (early: $127,379, late: $121,049). The incidence of repeat surgery at the index level within 12 months did not differ based on the timing of surgery (early 5% vs. late 7%).

Comparison based on surgical approaches

Median LOS was longer following circumferential procedure (early surgery: 10 days, late surgery: 13 days) compared to those who underwent anterior-only (early surgery: six days, late surgery: eight days) or posterior-only (early surgery: seven days, late surgery: nine days) procedures. Similarly, more than half of the patients were discharged home following anterior-only procedures in both cohorts. Complications were higher in the circumferential surgery cohort compared to anterior-only and posterior-only cohort in patients undergoing either early or late surgery for AcSCI. Interestingly, payments during index hospitalization and combined payments (index + 12 months) were higher only for early circumferential cohort compared to early anterior-only/posterior-only cohorts. This difference was not seen in the late surgery cohort. Patients in early circumferential and posterior-only surgical approaches incurred higher number of ER re-admissions within 30-days post-procedure and hospital admissions (12 months post-discharge) compared to anterior-only approaches. However, such a difference was not seen in the late surgical cohort. Similarly patients who underwent circumferential and posterior-only surgical approaches incurred a higher number of outpatient services compared to anterior-only procedures in both early and late surgical cohorts (Tables 4-5).

**Table 4:** Outcomes comparisons during the acute phase (first 12 months of SCI).

Outcomes					Early sx					Late sx (after 1 day)					Early sx Total vs Late sx Total
					Early sx Total	Anterior	Posterior	Circumferential	p-value	Late sx Total	Anterior	Posterior	Circumferential	p-value	
					n=1299	n=715	n=322	n=262		n=305	n=178	n=79	n=48		p-value
Index hospitalization															
		Length of stay, median (IQR)			7 (4, 16)	6 (3, 16)	7 (4, 13)	10 (5, 19)	.	9 (5, 17)	8 (5, 17)	9 (5, 14)	13 (7, 19)	0.0416	.
		Payment, median (IQR)			88105 (38880, 211680)	77605 (36266, 185711)	74622 (34500, 204999)	124803 (62549, 271134)	.	88003 (38565, 190191)	81926 (33588, 182686)	79727 (34251, 178967)	113631 (59062, 253918)	0.1763	0.626
		Discharge home, n (%)			612 (47%)	368 (52%)	145 (45%)	99 (38%)	0.0005	167 (55%)	109 (62%)	41 (52%)	17 (35%)	0.0044	0.016
		Complications, n (%)			658 (51%)	337 (47%)	165 (51%)	156 (60%)	0.0026	150 (49%)	80 (45%)	39 (49%)	31 (65%)	0.054	0.6431
30-days post discharge															
		ER admission, n (%)			142 (11%)	63 (9%)	44 (14%)	35 (13%)	0.0253	35 (11%)	21 (12%)	10 (13%)	4 (8%)	0.7432	0.785
		Complications, n (%)			316 (24%)	162 (23%)	82 (25%)	72 (27%)	0.256	78 (26%)	40 (22%)	21 (27%)	17 (35%)	0.184	0.6488
12-month post discharge															
	ER admissions, n (%)				525 (40%)	280 (39%)	129 (40%)	116 (44%)	0.3491	120 (39%)	63 (35%)	30 (38%)	27 (56%)	0.0306	0.7313
	Hospital admissions														
			Admitted, n (%)		457 (35%)	242 (34%)	118 (37%)	97 (37%)	0.5349	123 (40%)	64 (36%)	38 (48%)	21 (44%)	0.1627	0.0923
			# Readmissions, median (IQR)		0 (0, 1)	0 (0, 1)	0 (0, 1)	0 (0, 1)	0.2735	0 (0, 1)	0 (0, 1)	0 (0, 1)	0 (0, 2)	0.2098	0.09
			Payments, median (IQR)		0 (0, 21148)	0 (0, 18240)	0 (0, 21148)	0 (0, 34486)	0.3071	0 (0, 26965)	0 (0, 13993)	0 (0, 32398)	0 (0, 64591)	0.1129	0.1851
	Outpatient services														
				# Services, median (IQR)	117 (47, 242)	101 (42, 223)	130 (49, 256)	153 (66, 302)	.	100 (38, 210)	89 (33, 183)	107 (46, 210)	128 (60, 240)	0.1281	0.0125
				Payments, median (IQR)	13801 (4624, 44543)	12109 (3981, 35980)	13873 (4906, 45418)	21002 (7011, 58244)	.	12635 (3524, 34447)	12101 (2437, 26849)	14150 (4073, 38850)	17837 (7583, 41842)	0.05	0.1367
	Medication refills														
			# refills, median (IQR)		23 (3, 49)	21 (2, 47)	25 (4, 52)	28 (3, 55)	0.1766	24 (5, 58)	20 (4, 50)	24 (5, 66)	39 (12, 59)	0.1192	0.1974
			Payments, median (IQR)		897 (13, 3511)	821 (11, 3248)	909 (13, 3831)	1298 (13, 3778)	0.2963	1263 (53, 4139)	898 (22, 3898)	2031 (102, 4347)	2008 (200, 4615)	0.342	0.096
	Combined payments, median (IQR)				23885 (6647, 79417)	19926 (5413, 71099)	25128 (7800, 78615)	37922 (9602, 115954)	.	20245 (6387, 83528)	15881 (4085, 75827)	31874 (7285, 70974)	31649 (10928, 119134)	0.0733	0.5334
Cumulative payment: Index + 12-month, median (IQR)					127379 (57214, 301910)	109977 (50662, 281427)	121236 (54159, 294903)	195990 (87371, 408397)	.	121049 (58675, 281853)	109917 (53836, 261442)	121049 (58903, 228581)	181779 (74939, 387061)	0.094	0.5891
12-month Repeat surgery, n (%)					60 (5%)	33 (5%)	15 (5%)	12 (5%)	0.999	21 (7%)	13 (7%)	7 (9%)	1 (2%)	0.3237	0.1038

**Table 5:** Multivariable adjusted outcomes comparisons during the acute phase (first 12 months of SCI).

			Early sx					Late sx		
			Anterior	Posterior		Circumferential		Anterior	Posterior	Circumferential
Variable			n=715	n=322		n=262		n=178	n=79	n=48
Index hospitalization										
	Length of stay		Reference	0.795 (0.643, 0.983)		0.946 (0.787, 1.137)		Reference	16.384 (6.439, 41.69)	2.378 (0.769, 7.356)
	Payment		Reference	1.024 (0.868, 1.208)		1.229 (1.066, 1.418)		Reference	1.462 (0.999, 2.139)	1.153 (0.739, 1.8)
	Discharge home		Reference	0.805 (0.609, 1.064)		0.62 (0.459, 0.838)		Reference	0.76 (0.434, 1.334)	0.343 (0.174, 0.68)
	Complications		Reference	1.203 (0.909, 1.591)		1.518 (1.123, 2.052)		Reference	1.133 (0.647, 1.984)	2.17 (1.095, 4.299)
30-days post discharge										
	ER admission		Reference	1.861 (1.215, 2.852)		1.591 (1.012, 2.503)		Reference	1.4 (0.567, 3.46)	0.528 (0.159, 1.748)
	Complications		Reference	1.047 (0.761, 1.441)		1.199 (0.86, 1.67)		Reference	1.096 (0.575, 2.089)	1.781 (0.868, 3.655)
12-month post discharge										
	ER admissions		Reference	1.34 (0.976, 1.838)		1.164 (0.842, 1.609)		Reference	1.904 (0.98, 3.698)	2.559 (1.205, 5.432)
	Hospital admissions									
		Admitted	Reference	1.15 (0.863, 1.531)		1.069 (0.789, 1.449)		Reference	1.618 (0.921, 2.841)	1.252 (0.641, 2.445)
		# Readmissions	Reference	1.176 (1.012, 1.367)		1.24 (1.065, 1.444)		Reference	1.09 (0.817, 1.456)	1.331 (0.981, 1.805)
		Payments	Reference	0.832 (0.551, 1.254)		0.663 (0.429, 1.025)		Reference	0.091 (0.003, 2.677)	0.141 (0.012, 1.671)
	Outpatient services									
		# Services	Reference	1.17 (1.158, 1.182)		1.293 (1.28, 1.307)		Reference	1.126 (1.1, 1.152)	1.252 (1.22, 1.284)
		Payments	Reference	1.259 (1.03, 1.54)		1.407 (1.167, 1.697)		Reference	1.21 (0.652, 2.244)	0.788 (0.321, 1.934)
	Medication refills									
		# Refills	Reference	1.125 (1.1, 1.151)		1.184 (1.157, 1.212)		Reference	1.174 (1.122, 1.227)	1.221 (1.16, 1.285)
		Payments	Reference	1.254 (0.951, 1.654)		1.4 (1.054, 1.859)		Reference	1.01 (0.684, 1.492)	1.012 (0.621, 1.65)
	Combined payments		Reference	1.067 (0.838, 1.358)		1.003 (0.789, 1.275)		Reference	1.014 (0.539, 1.907)	1.085 (0.577, 2.041)
Cumulative payment: Index + 12-month			Reference	1.046 (0.891, 1.228)		1.166 (1.01, 1.347)		Reference	1.297 (0.876, 1.92)	1.123 (0.723, 1.743)
12-month Repeat surgery			Reference	1.034 (0.548, 1.953)		0.991 (0.502, 1.959)		Reference	1.165 (0.421, 3.22)	0.303 (0.037, 2.465)
Multivariable regression are done for each outcome with										
Demographics (Age, Gender and Insurance, Elixhauser index) and early/late/approach group										

Overall, combined median payment (index hospitalization + 12 months) was significantly higher for early circumferential cohorts compared to anterior or posterior-only cohort ($195,990 and $109,977 vs. $121,236 respectively) (Tables 4-5; Figure 3). Interestingly, we did not find differences in the incidence of repeat cervical spine surgery (12 months following index surgery) among anterior (early 5%, late 7%), posterior (early 5%, late 9%) and circumferential (early 5%, late 2%) groups. This difference did not reach significance on multivariate analysis as well (Tables 4-5).

## Discussion

Based on the MarketScan database, we found that posterior cervical decompression and fusion for AcSCI are increasingly being performed for AcSCI. However, the exact reason for this trend cannot be determined using the MarketScan database without clinical information regarding decision-making in these patients with SCI. There was no difference in healthcare utilization during index hospitalization and combined payments based on the timing of surgery (early vs. late). Patients who underwent late surgeries (>24 hrs) were likely to be discharged home and had a higher number of readmissions during 12 months following discharge compared to those who had early surgery (<24 hrs). There was no difference in terms of complications (during index hospitalization/30-days post-discharge) and repeat surgeries during 12 months following discharge based on the timing of surgery (early vs. late).

Although the circumferential procedure was associated with increased health care utilization at index hospitalization and overall payments (combined index and 12 months) compared to the anterior-only procedure, this difference was seen only in patients who underwent early surgeries. Also, late circumferential surgeries were associated with higher complications (OR 2.17; 95% CI 1.095 - 4.299) compared to those who underwent early circumferential surgeries (OR 1.518; 95% CI 1.123 - 2.052). This difference may be attributed to the small number of patients in the late surgery cohort. Also, circumferential procedures are usually performed for severe cervical spine injuries with gross instability and mal-alignment causing compression of neural elements. Therefore, these procedures are associated with high complications and initial higher health care costs compared to anterior or posterior-only procedures. To our knowledge, the present study is the first to analyze healthcare utilization with regard to the timing of surgery and surgical approaches for AcSCI.

Mac-Thiong et al. [11] investigated the impact of timing and type of surgical approaches in patients with SCI, using their institutional database. In this study, the authors included patients (n=477) with both cervical (48%) and thoracic/lumbar (52%) spinal cord injury. Interestingly, 80% (384/477) of patients had surgery > 24 hrs after presentation in this study, in contrast, 81% of patients had surgery <24 hrs in our study. The above-mentioned study [11] showed that hospitalization costs were lower in patients who underwent early surgery compared to those who underwent late surgery (mean Canadian $20,525 vs $25,036). However, there was no difference in the cost based on the type of surgical approaches (anterior vs posterior vs combined). In contrast, our study showed that there was no difference in index hospitalization payment based on the timing of surgery, however, circumferential procedures were associated with higher cost compared to anterior or posterior approaches only at both index hospitalization and combined (index + 12 months) time-line. This may be attributed to different sample populations (only cervical spinal cord in our study), geographical distribution, practice patterns and retrospective data from a single institution.

Healthcare utilization and acute traumatic spinal cord injury

Costs related to SCI amount to approximately $9.7 billion annually according to some reports [21]. Previous literature has described differences in healthcare payments based on location and injury severity. In a Veterans Hospitals' study, patrons with complete cervical injury accounted for $28,334 annual payments when compared to those with incomplete thoracic injury totaling $16,792 [22]. Additionally, distinctions have been made regarding healthcare payments for those with persistent neuropathic pain after SCI, showing $22,545 additional incremental payments for those with neuropathic pain compared with those who did not report neuropathic pain after injury [23]. Further, inpatient visits and emergency visits were also higher for those who experienced neuropathic pain after injury in the 12 months following injury. 

With decreasing incidence of mortality from SCI and increasing incidence of incomplete neurological injury including central cord syndrome in elderly patients, the health care costs related to the care of these patients have a huge impact on the health system [3, 24-26]. A retrospective study from Level 1 trauma registry showed that 58% of patients required additional inpatients or emergency health care utilization during the first year after injury [26]. Similarly, we found that 40% and 35% of patients in the early surgery group (late surgery 39% and 40% respectively) required ER admissions and hospital re-admissions respectively during the 12 months following discharge.

A population-based cohort study showed that the attributable costs in the first year following SCI was estimated to be $121,600 per person (2002 Canadian dollars) for complete SCI and $42,100 per person for incomplete SCI with annual follow-up costs of $5,400 and $2,800 per persons respectively [25]. Another study showed that total cumulative charges (index hospitalization and readmissions during 12 months after discharge) increased from $102,900 per person (2005 Canadian dollars) in 2003-2004 to $123,674 in 2005-2006. This study found that the largest cost driver in their health system was inpatient rehabilitation. Another study estimated the direct cost incurred by performing a comprehensive survey of US SCI patients [27]. Based on the 1988 US dollar value, this study showed that the mean initial hospitalization costs were $95,203, followed by $2,958 per year for re-hospitalization and $4,908 per year for medical services. Also, home modification costs may exceed $8,000 in these patients [27]. Similarly, in our study we found that, total median cumulative costs (index + 12 months follow-up) in patients who underwent early surgical decompression and fusion for AcSCI was $127,379 (2016 US dollars, range 57,214- 301,910) compared to $121,049 (range 58,675 - 281,853) in late surgery group. 

Interestingly, we did not find differences between late surgical approaches in terms of payments (index and 12 months follow-up), however, there were differences in complication rates and discharge dispositions. We hypothesized that patients who underwent late surgical procedures were likely to have some mild spinal cord injury with no gross instability, however, such information cannot be extracted from this database. Also, this difference may be attributed to the small number of patients in the late circumferential group (n=48). However, when we compared different surgical groups irrespective of the timing of surgery, the circumferential group was associated with higher payments (index and combined) compared to anterior-only procedures. 

Limitations

A major limitation of this database is that it does not provide clinical information related to individual patients, which may have guided the treating physician in selecting the optimal surgical approach for a given patient. Therefore, selection bias cannot be eliminated using this database. Also, the extent and appropriateness of surgical decompression and extent of fusion cannot be extracted, which may have caused reported differences. The data is extracted using ICD and CPT billing codes. Therefore, there is a potential for coding error, albeit small. Complications specific to the procedure and quality of life data cannot be extracted using this database. Due to these limitations, these results need to be carefully interpreted. However, the MarketScan database provides a comprehensive overview of clinical problems throughout the United States. This database provides an opportunity to study national trends over the years. One unique advantage of the MarketScan database is that it provides longitudinal clinical follow-up with a number of clinical services (outpatient visits, medication refills etc.) received during the period and corresponding healthcare utilization. Thus, this database provides a complete understanding of the management outcomes and healthcare burden instead of a cross-sectional view of the clinical problem. Our study provides insight into the impact of surgical timing and different surgical approaches with regards to healthcare utilization in patients undergoing surgery for traumatic AcSCI.

## Conclusions

Late surgeries were associated with a higher likelihood to be discharged home, lower utilization of outpatient services and no difference in payments (index and cumulative) compared to early surgeries. Circumferential approaches were associated with higher complication rates, less likelihood to be discharged home, higher utilization of outpatient services compared to anterior-only approaches. However, associated increased payments (index and cumulative) were noted only in patients who underwent early circumferential procedures. These findings can be useful for both clinicians and policymakers to guide decision-making and to reduce the overall health care burden related to AcSCI.
